# Assessment of the Efficacy and Safety of Acalabrutinib in Chronic Lymphocytic Leukemia (CLL): A Systematic Review and Meta‑Analysis 

**DOI:** 10.7759/cureus.70259

**Published:** 2024-09-26

**Authors:** Rashad Q Othman

**Affiliations:** 1 Pathology, Northern Border University, Faculty of Medicine, Arar, SAU

**Keywords:** acalabrutinib, bruton’s tyrosine kinase inhibitor, chronic lymphoid leukemia, drug efficacy, drug safety

## Abstract

Chronic lymphoid leukemia (CLL) is a common adult leukemia that has been treated with chemoimmunotherapy, which has significant toxicity among patients. Advances in CLL understanding have led to targeted therapies, such as Bruton's tyrosine kinase (BTK) inhibitors. Acalabrutinib is one of the second-generation BTK inhibitors and offers improved selectivity, reducing off-target effects. This systematic review and meta-analysis analyze data from multiple clinical trials to assess acalabrutinib efficacy and safety among patients with CLL. A literature search was carried out in PubMed, Cochrane Controlled Register of Trials (CENTRAL), MEDLINE (Medical Literature Analysis and Retrieval System Online), and Ovid databases for articles published until 2024. The outcomes included the overall response rate (ORR), complete response rate (CRR), 24-month progression-free survival rate, and grade ≥3 adverse events (AEs). Meta-analysis was performed using jamovi software. The search strategy yielded 823 records. After assessing the eligibility of the retrieved studies, the systematic review finally included six clinical trials, including 808 patients. The findings demonstrated significant efficacy of acalabrutinib, with a pooled ORR (P < 0.001) and a pooled CRR (P = 0.001). The pooled 24-month progression-free survival (PFS) rate showed a significant improvement in maintaining patient safety and treatment effectiveness (P <0.001). However, hematological AEs such as neutropenia, anemia, and thrombocytopenia were reported across studies. The pooled grade ≥ 3 AEs rate was 0.51 (95% CI: 0.21-0.81, I² = 98.21%, P <0.001), indicating a notable incidence of severe side effects. Acalabrutinib is an active drug in treating CLL that induces significant clinical benefits concerning response rates and PFS. However, acalabrutinib should be managed carefully to mitigate the risk of severe AEs. Further trials should be focused on assessing and modulating the safety of acalabrutinib.

## Introduction and background

Leukemia is a type of cancer characterized by leukocyte production, an abnormal process that can be of primary or secondary origin. It is categorized into either acute or chronic based on the rapidity of proliferation. In addition, it can be classified into myeloid or lymphoid, depending on the origin of the cells. There are different types of leukemia. The main types include acute myeloid leukemia (AML), chronic myeloid leukemia (CML), acute lymphoid leukemia (ACL), and chronic lymphoid leukemia (CLL). While myeloid leukemia involves myeloid lineage, lymphoid leukemia involves the lymphoid chain [1]. Notably, in 2018, leukemia was the 15th most prevalent diagnosed cancer worldwide, as it accounted for 437,033 incident cancer individuals and 309,006 cancer deaths [2].

CLL represents one of the most common forms of adult leukemia, characterized by the clonal proliferation of a type of B lymphocyte. Although its course usually has a slow and indolent evolution, it can sometimes become very progressive and require active treatments. Conventionally, the management of CLL has primarily focused on chemoimmunotherapy, which has shown to be effective. However, it is still associated with significant toxicity and suboptimal outcomes in specific patient groups. Recently, advances in understanding CLL pathophysiology have resulted in the development of targeted therapies, which have changed the treatment approach and significantly enhanced patient clinical outcomes [3].

One essential targeted therapy that has gained wide attention is Bruton's tyrosine kinase (BTK). BTK inhibitors have been considered necessary in treatment plans due to their significant role in the signaling of B-cell receptors. For instance, Ibrutinib, which was the first BTK inhibitor approved for CLL, showed a profound efficacy. However, it was associated with several undesirable off-target effects. This drawback set the stage for researchers to develop second-generation BTK inhibitors to solve this issue. Among the second-generation BTK inhibitors, acalabrutinib was developed to be more selective in its inhibition of BTK compared to the first-generation inhibitors, thus reducing the incidence of off-target effects [4,5].

Several clinical trials have taken the potential anti-leukemic activity of acalabrutinib as a focus and compared its safety profile to ibrutinib. For instance, the ELEVATE-RR trial showed that acalabrutinib has less risk of atrial fibrillation and other adverse effects while being effective compared to ibrutinib. These findings have established the role of acalabrutinib as a viable treatment for an increased number of CLL patients, especially those who have a high risk for cardiovascular complications [6,7].

European Medicines Agency (EMA) and the United States Food and Drug Administration (FDA) approved acalabrutinib to treat CLL, relying on clinical trial data. This indicates that the data should be monitored during commercialization. In clinical studies, acalabrutinib significantly prolonged progression-free survival (PFS) and improved overall response rate (ORR) in patients with CLL. In addition to clinical trial data, new real-world evidence has further demonstrated the safety and efficacy of acalabrutinib in patients with several different characteristics, including those who may not be able to tolerate available alternative options [8,9].

As increasingly large amounts of data point to the effectiveness of acalabrutinib, its efficacy and safety profile in treating CLL need to be studied thoroughly. Some clinical studies have evaluated the therapeutic efficacy of acalabrutinib. Therefore, this systematic review and meta-analysis aimed to analyze data from multiple clinical trials assessing its efficacy and safety among patients with CLL.

## Review

Methods

Methodology

The study was conducted in alignment with the guidelines outlined in the Cochrane Handbook for Systematic Reviews of Interventions, version 6, and the findings were reported following the Preferred Reporting Items for Systematic Reviews and Meta-Analyses (PRISMA) standards [10].

Eligibility Criteria

Inclusion criteria: The inclusion criteria were (i) prospective clinical studies including single-arm studies and RCTs, (ii) studies including patients diagnosed with CLL, (iii) studies involving patients treated with acalabrutinib, either as a monotherapy or in combination with other medications, (iv) studies that reported both efficacy and safety outcomes, including the overall response rate (ORR), complete response (CRR), and grade ≥3 adverse events (AEs). Only studies published in English were eligible. 

Exclusion criteria: The exclusion criteria were (i) Patients didn't all get the same dose, (ii) Significant errors in statistical methodologies, (iii) Duplicate studies, (iv) incomplete reported results, (v) The type of articles, such as when conference summaries, letters, reviews, or just a report about one patient, (vi) studies that didn't include all the information, and (vii) non-human studies only on cells or animals.

Search Strategy

We conducted a comprehensive search through five databases: PubMed, Cochrane Central Register of Controlled Trials (CENTRAL), MEDLINE (Medical Literature Analysis and Retrieval System Online), Ovid, and Scopus, up to July 10, 2024. The search was initiated on July 10, 2024, using the following query syntax tailored to each database. In PubMed, the search terms used were ("Leukemia" OR "Chronic Lymphocytic Leukemia" OR "CLL") AND "acalabrutinib." The same syntax was applied to CENTRAL, MEDLINE, Ovid, and Scopus, ensuring consistency across platforms. The search strategy was designed to retrieve studies relevant to the topic while adapting to the specific search capabilities of each database. Aiming for a thorough exploration, examining all likely applicable studies won't have geographical, racial, or age constraints. Further, detailed proofreading of references listed in retrieved articles and re-marks was diligently conducted.

Selection of Studies

The processes of online search, screening the titles and abstracts, as well as screening the full text of relevant articles were conducted by the author. 

Data Extraction

All relevant studies were downloaded on Mendeley (Mendeley Ltd., London, United Kingdom), and duplicates were removed. The extracted data according to the inclusion and exclusion criteria was performed using a self-prepared data collection form. The following data was extracted from each study: general characteristics of each study such as author’s name, publication year, and phase of the clinical trial, and descriptive data of the patients, including sample size, median age, and disease condition, in addition to the acalabrutinib dose used. The primary endpoints were ORR and CRR. The secondary endpoints included a 24-month PFS rate, and the number of grade ≥3 AEs that were reported in the studies.

Measured Outcomes

Primary outcome: The ORR and CRR were the primary outcomes. The ORR is defined as the proportion of patients who achieve either a complete response or a partial response to treatment. It includes all patients who show a measurable reduction in disease burden, encompassing both complete and partial responses. CRR refers to the complete disappearance of all detectable clinical and laboratory evidence of disease. This includes the normalization of blood counts and the absence of lymphadenopathy, splenomegaly, and hepatomegaly, along with the resolution of any bone marrow involvement. The CRR specifically represents the proportion of patients who achieve this complete resolution of disease [11].

Secondary outcome: The 24-month PFS and the number of grade ≥3 AEs were the secondary outcomes. PFS is defined as the time from randomization until the first evidence of disease progression or death. Grade ≥3 AEs refer to severe or life-threatening side effects or complications that occur during a clinical trial or treatment [12].

Assessment of the Quality of the Included Studies

The risk of bias (ROB) was assessed using the Cochrane revised version of the ROB tool for randomized clinical trials (RCTs) [13]. The ROB2 tool includes five domains: randomization, deviations from the assigned intervention, missing outcome data, measurement of the outcome, and selective reporting of the results. Moreover, the overall ROB is evaluated by selecting the highest level of ROB out of the five domains in each study. The Robvis tool was used to visualize the figures [14]. The methodological index for non-randomized studies (MINORS) was conducted to evaluate prospective single-arm studies [15].

Data Synthesis

All data analyses were conducted using the Jamovi software [16]. For acalabrutinib efficacy, the pooled ORR and CRR were calculated along with 95% confidence intervals (CI). To assess heterogeneity across studies, Cochrane’s Q chi-square test and I² statistic were employed to quantify the level of variation among the study results. A p-value < 0.05 was considered statistically significant. For safety, we measured the number of grade ≥3 AEs with 95% CI. The restricted maximum likelihood model was used in the meta-analysis.

Results

Results of Literature Search and Study Selection

**Figure 1:**
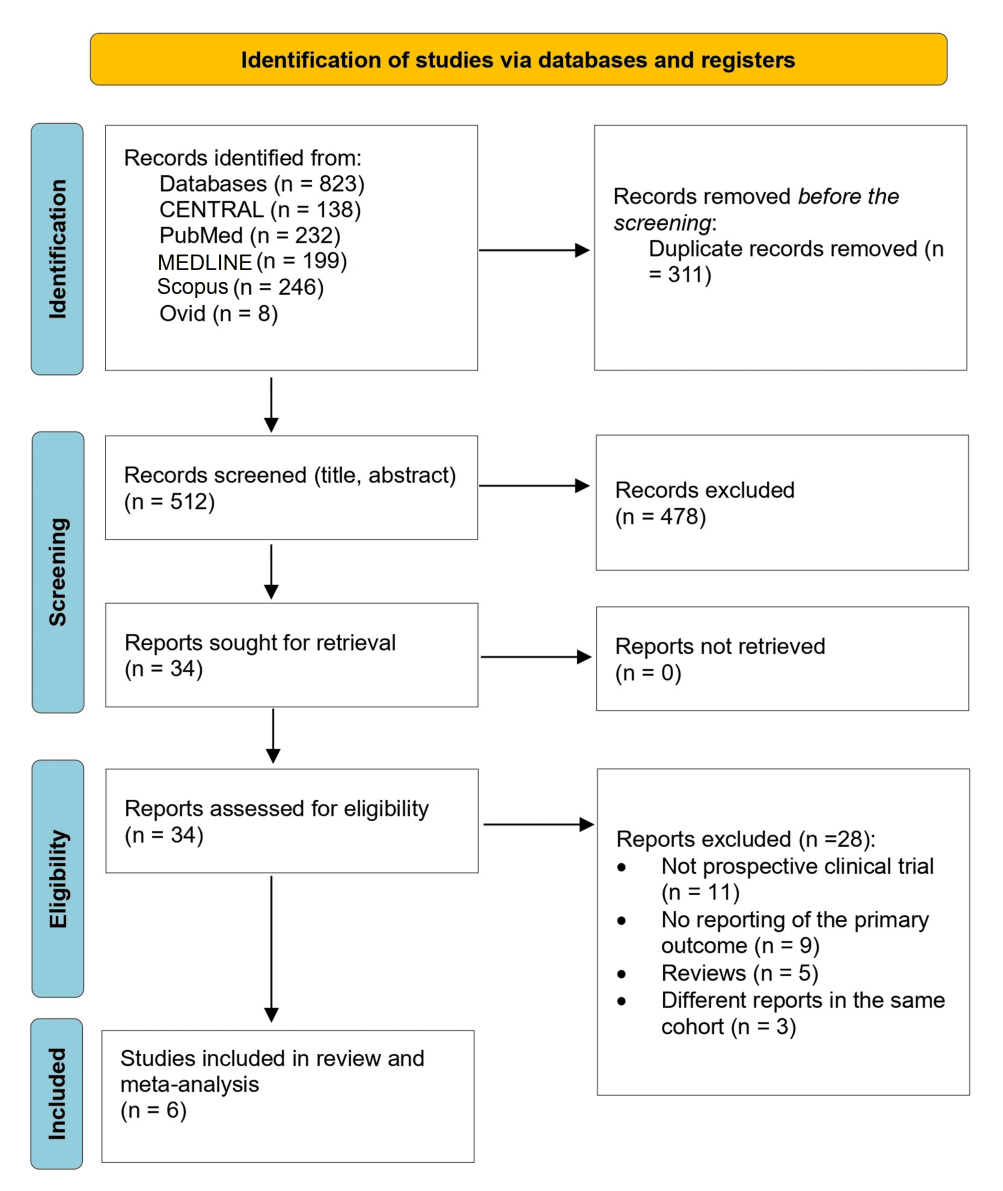
The PRISMA flowchart of the selection process in the review PRISMA: Preferred Reporting Items for Systematic Reviews and Meta-Analyses; MEDLINE: Medical Literature Analysis and Retrieval System Online; CENTRAL: Cochrane Controlled Register of Trials

The search strategy yielded 823 records, of which 311 duplicates were removed. The remaining 512 articles underwent screening of their titles and abstracts, and 478 were excluded. The remaining 34 records were retrieved for full-text screening and were assessed for eligibility. Of these, 28 records were excluded. Finally, six studies were eligible for inclusion in the present review [17-22]. Figure 1 illustrates the details of the study selection process. The meta-analysis assessed the efficacy and safety of acalabrutinib for 808 CLL patients across four single-arm studies [17,19,21,22], and two-phase three randomized studies [18,20]. Since some records have more than one disease state or intervention, we considered only acalabrutinib monotherapy as an intervention. One study included two different dosing regimens of acalabrutinib [21]. Four studies were conducted in the United States [17,21,23,24], and two were conducted across different centers in different countries [20,22]. The participants' ages ranged from 33 to 89 years across studies. The studies were conducted between 2019 and 2021. The median follow-up duration ranged between 18 and 53 months. Table 1 presents the main characteristics of the included studies. 

**Table 1:** Characteristics of the included trials TN-CLL: Treatment-Naive Chronic Lymphoid Leukemia; CLL: Chronic Lymphoid Leukemia; R/R CLL: Relapsed/Refractory Chronic Lymphoid Leukemia; NA: Not Available; IQR: Interquartile Range

Study	Study type	Diseases condition	Intervention	Sample size	Median age (range), years	Median follow-up duration (months)
Byrd et al., 2021 [17]	Phase I/II	TN-CLL	Acalabrutinib (100 mg) twice daily	99	64 (33–85)	53
Byrd et al., 2021 [18]	Phase III	Previously treated CLL	Acalabrutinib (100 mg) twice daily	268	66 (41–89)	40.9
Rogers et al., 2021 [19]	Phase II	Ibrutinib intolerant R/R CLL	Acalabrutinib (100 mg) twice daily	179	70	28.3 (IQR 25.6–33.1)
Sharman et al., 2020 [20]	Phase III	TN-CLL	Acalabrutinib (100 mg) twice daily	179	70	28.3 (IQR 25.6–33.1)
Sun et al., 2020 [21]	Phase II	TN/RR CLL	Acalabrutinib (100 mg) twice daily	24	64 (45–83)	NA
			Acalabrutinib (200 mg) once daily	24		
Awan et al., 2019 [22]	Phase I/II	Ibrutinib-intolerant CLL	Acalabrutinib (100 mg) twice daily	35	64 (50–82)	Safety: 18.5 Efficacy: 19

ORR and CRR

**Figure 2:**
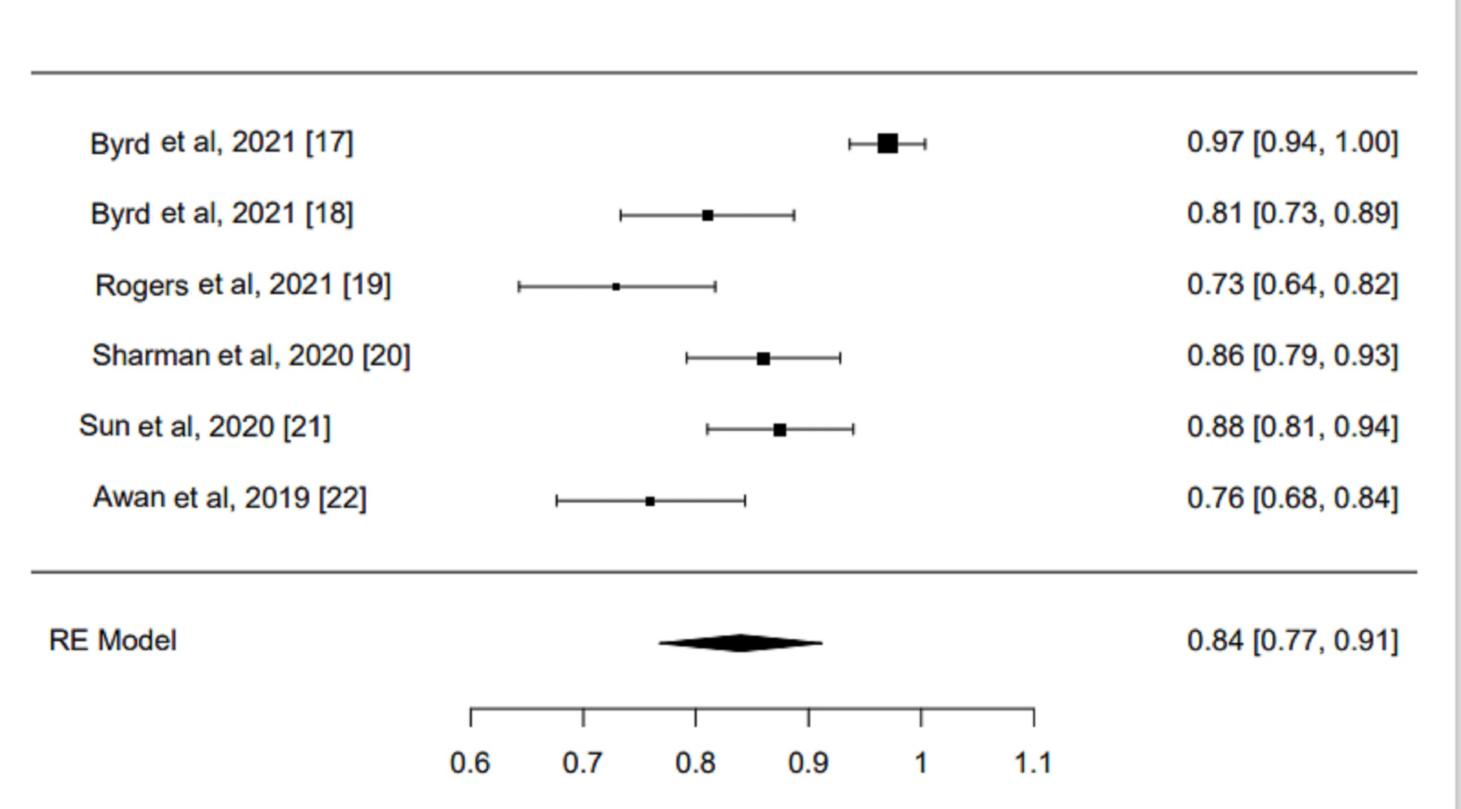
ORR analysis of the included studies ORR: Overall Response Rate; RE Model: Random Effects Model References: [17-22]

**Figure 3:**
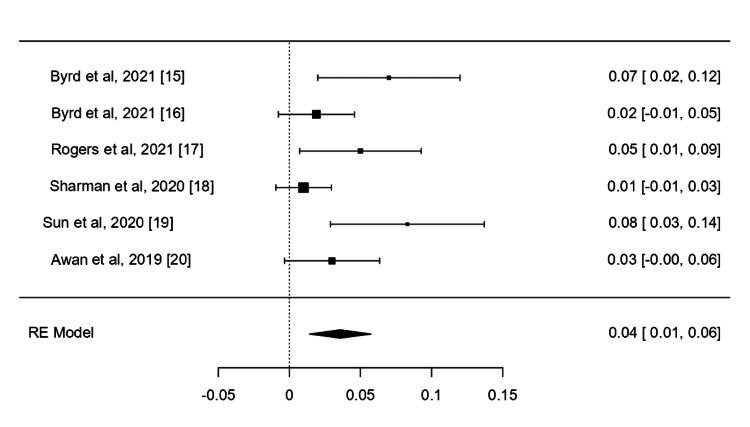
CRR of the included studies CRR: Complete Response Rate; RW: Random Effect References: [17-22]

All the six included trials reported ORR and CRR as primary outcomes. The pooled ORR for acalabrutinib was 0.84 (95%CI, 0.77-0.91, I^2^=86.74%, P=0<001), while the pooled CRR was 0.04 (95%CI, 0.01-0.06, I²=57.78%, P=0.001). There was high significance in both reported outcomes. Analysis was conducted using a restricted maximum likelihood model, which confirmed the considerable efficacy of acalabrutinib treatment in CLL (Figures 2-3).

The 24-Month PFS

**Figure 4:**
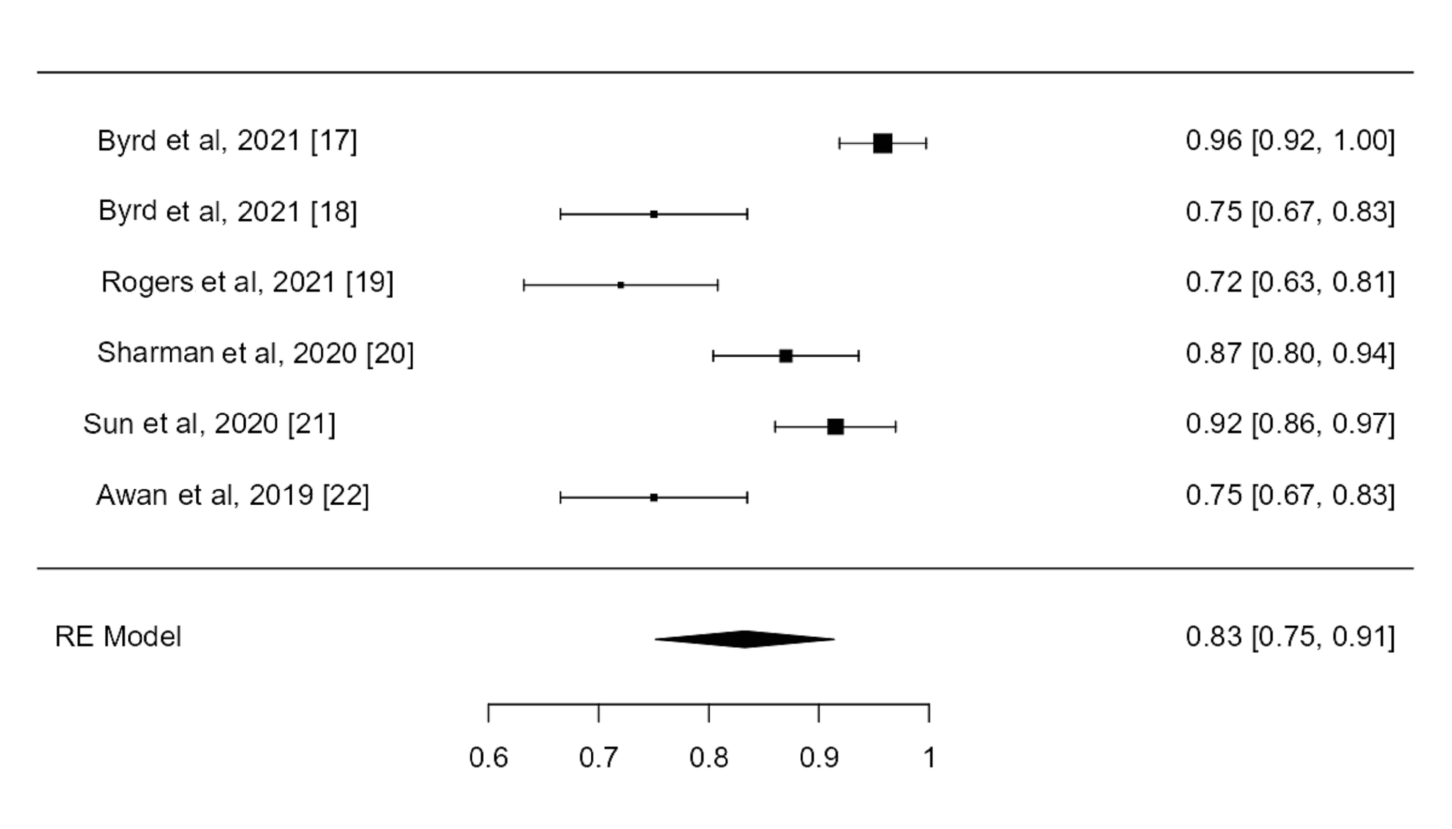
PFS of the included studies PFS: Progression-Free Survival; RW: Random Effect References: [17-22]

All included studies reported PFS data for 24 months. The pooled 24-month PFS rate of CLL patients managed by acalabrutinib was 0.83 (95%CI, 0.75-0.91, I^2^ =87.49%, P<001). The results were statistically significant indicating that the intervention resulted in considerable improvement of patients maintaining their safety (Figure 4).

AE *Grade *≥3

**Figure 5:**
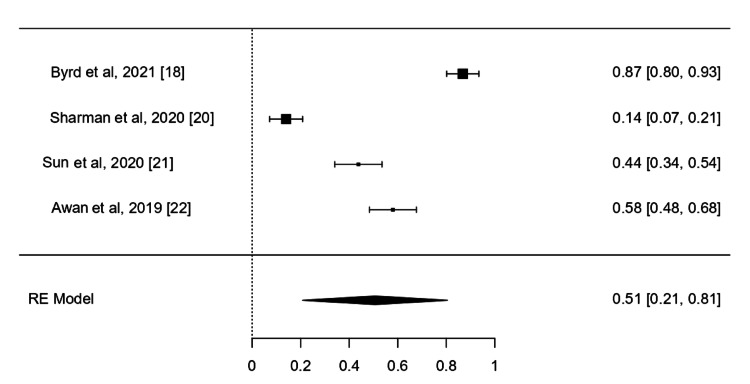
Grade ≥3 AE of the included studies AE: Adverse Event; RE: Random Effect References: [18,20-22]

AEs were reported in all studies; neutropenia, thrombocytopenia, and anemia were the main hematological AEs. The pooled rate of grade ≥3 of AE was reported in four studies. The pooled analysis was 0.51 (95%CI, 0.21-0.81, I^2^ =98.21%, P <001) (Figure 5).

ROB Assessment

**Figure 6:**
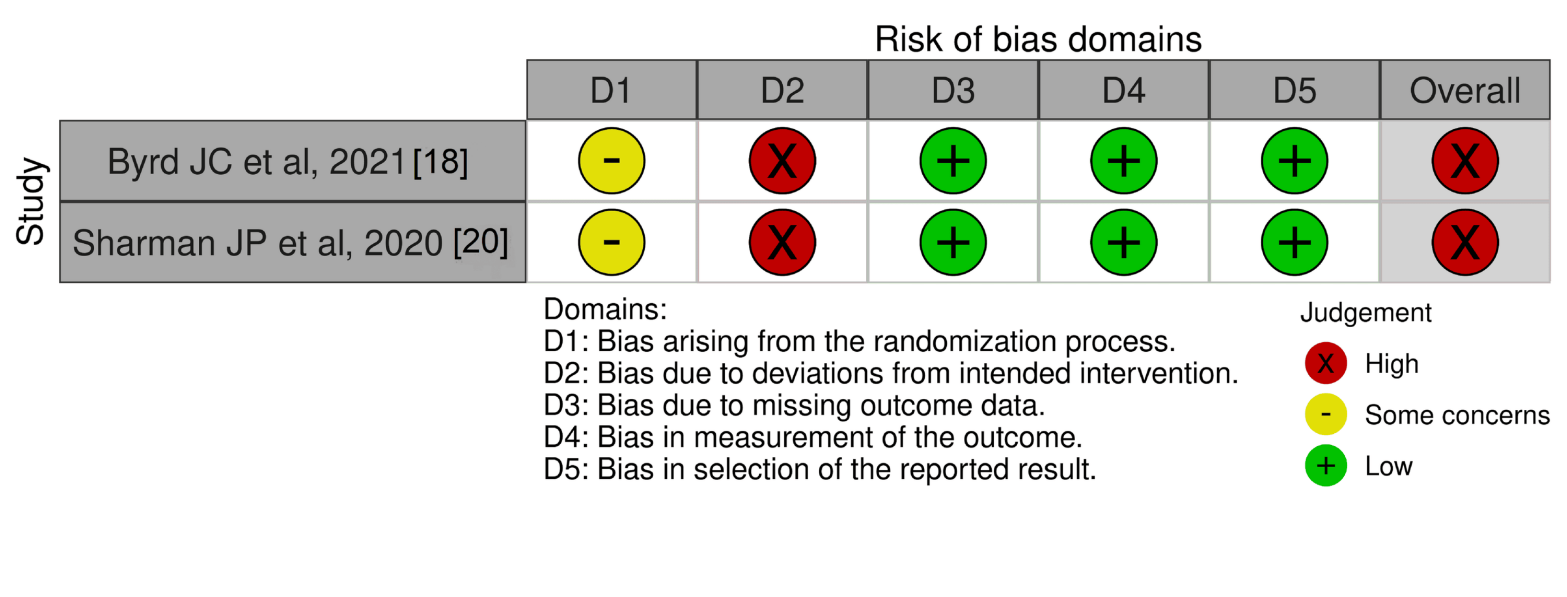
Risk of bias assessment of the included trials (traffic light plot) References: [18,20]

**Figure 7:**
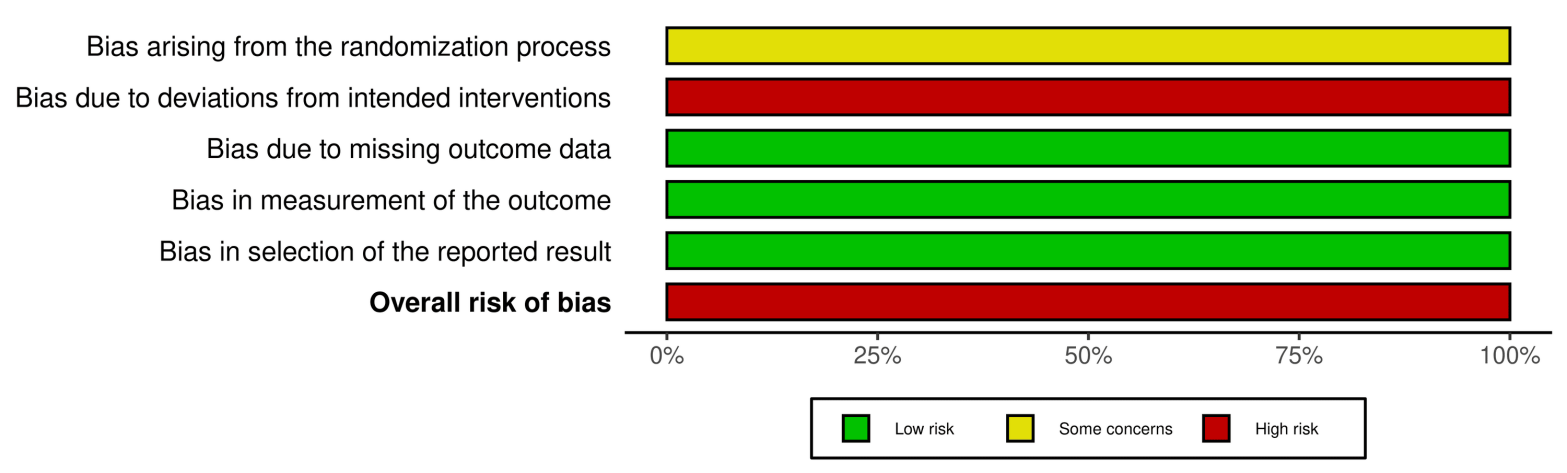
Risk of bias summary

Four single-arm studies were assessed using the Methodological Index for Non-Randomized Studies (MINORS) score. Each criterion is scored from 0 to 2, with higher scores indicating a lower risk of bias. Non-comparative studies have a maximum score of 16, while comparative studies can score up to 24. The score ranged from 11 to 20 points, which could be considered acceptable for the present meta-analysis (Table 2). Two RCTs were assessed for quality using the ROB2 tool (Figures 6-7).

**Table 2:** Quality assessment of included single-arm studies (I): A clearly stated aim; (II): Inclusion of consecutive patients; (III): A prospective collection of data; (IV): Endpoints appropriate to the aim of the study; (V): An unbiased assessment of the study endpoint; (VI): Follow-up period appropriate to the aim of the study; (VII): Loss to follow-up less than 5; (VIII): Prospective calculation of the study size. For comparative studies, four additional criteria are included: (IX): An adequate control group; (X): contemporary groups; (XI): Baseline equivalence of groups, and (XII): Adequate statistical analyses.

Study	I	II	III	IV	V	VI	VII	VIII	IX	X	XI	XII	Total
Rogers et al, 2021 [17]	2	2	2	2	1	1	2	2	-	-	-	-	14
Byrd et al, 2021 [17]	2	2	2	2	1	1	2	2	-	-	-	-	14
Sun et al, 2020 [21]	2	2	2	2	1	1	1	1	2	2	2	2	20
Awan et al, 2019 [22]	2	2	2	2	1	1	1	0	-	-	-	-	11

Discussion

The present review updated the clinical information regarding the efficacy and safety profile of acalabrutinib in the management of CLL. The findings showed that acalabrutinib is a highly effective therapeutic intervention, especially among patients suffering from CLL. From this pooled result, ORR 0.84 (95%CI, 0.77-0.91) and CRR 0.04 (95%CI, 0.01-0.06), acalabrutinib achieved significant clinical response, which justifies trials that have established it as an important treatment for CLL.

Results for ORR and CRR presented were aligned with previous research, based on previous studies such as those by Byrd et al. (2016) [23], and Byrd et al. (2018) [24]. These studies confirmed that acalabrutinib has a high ORR in both treatment naïve and relapsed CLL patients, as the efficacy observed in our analysis might be explained through the mechanism of action of acalabrutinib as a selective BTK inhibitor. This targeted approach shows how acalabrutinib from earlier treatments is responsive to its effectiveness, especially in patients who do not respond well to other therapies.

Furthermore, our findings showed that the 24-month PFS is a significant endpoint in evaluating the long-term benefits of therapeutic interventions in CLL. A pooled rate of 0.84 (95%CI, 0.77-0.91) for 24-month PFS from our analysis indicates that many patients treated with acalabrutinib attain disease control for at least two years. This is consistent with the results of the ELEVATE-TN trial, which shows that single-agent acalabrutinib and acalabrutinib plus obinutuzumab significantly improve PFS versus chlorambucil plus obinutuzumab [6]. Moreover, the durable PFS observed in several studies confirms acalabrutinib's role in extending remission and delaying disease progression, which is a very important aspect of CLL management. The high PFS rate also indicates acalabrutinib’s ability to provide long-term benefits with continuous treatment, which is a significant advantage in CLL, where disease progression can result in high morbidity and mortality.

Interestingly, recent real-world studies and reviews have provided additional insights into the safety and efficacy of acalabrutinib. For instance, Yazdy et al. conducted a retrospective analysis highlighting the drug’s tolerability in a broader patient population, including those with extensive treatment histories and comorbidities [25]. Similarly, Delgado et al. focused on the importance of individualized patient management to optimize outcomes with acalabrutinib, particularly in terms of balancing the risk of AEs with the expected therapeutic benefits [8].

It is worth noting that referring to the ORR, high heterogeneity has been detected across the included trials, reflected by I² = 86.74%, which describes the range of patient demographics, disease stages, prior treatments, and study designs. Despite this heterogeneity, the results confirmed the efficacy of acalabrutinib, suggesting that the drug’s benefits are consistent across different patient groups. This broad applicability is particularly important given the heterogeneous nature of CLL itself, which often requires tailored treatment strategies.

Overall, acalabrutinib represents an important advancement in treating CLL based on the high response rates observed to date, with durable PFS and a generally manageable safety profile. However, AE management is still a major challenge, requiring a multidisciplinary approach if optimal outcomes for these patients are to be achieved. With acalabrutinib's continued integration into clinical practice, further refinement of its use, especially in specifying patient populations that derive the most benefit from this therapy and developing strategies to mitigate the risk of serious AEs, should be undertaken.

Future research may also provide combination therapies that could make acalabrutinib even more effective at a lower dose, hence reducing the risk of AEs. Biomarker-oriented studies to predict response or toxicity could offer personalized acalabrutinib therapy, therefore assuring that patients receive the most appropriate treatment according to their risk profile.

## Conclusions

Acalabrutinib is an effective treatment for CLL, demonstrating significant clinical benefits in terms of response rates and PFS. The current review found that both the pooled ORR and the pooled CRR for acalabrutinib were statistically significant, indicating a high level of efficacy. However, careful management is required to mitigate the risk of severe AEs. Therefore, further research on the optimal use of acalabrutinib, including combination therapies and personalized treatment strategies, is essential to maximize its therapeutic benefits while minimizing harm to patients.
